# Effects of Cryotherapy on Lower Leg Deep Tissue Temperature Changes: As Measured in Healthy Volunteers by Using MRI Technique

**DOI:** 10.7759/cureus.30384

**Published:** 2022-10-17

**Authors:** Yoshifumi Nanba, Saori Kawashima

**Affiliations:** 1 Department of Rehabilitation Science, Kobe University Graduate School of Health Sciences, Kobe, JPN

**Keywords:** lower leg, magnetic resonance imaging, t1 signal, deep tissue, intra-articular temperature, cryotherapy

## Abstract

Background: Since the 1950s, researchers have studied temperature changes in deep tissues caused by cooling stimuli. However, these changes have not been investigated non-invasively. Moreover, opinions are divided as to whether the temperature in the joints rises or falls. The present study investigated the effects of cooling on various tissues in the body, including muscles.

Methods: Seven healthy subjects (four males and three females) were enrolled (age, 21.5 ± 0.8 years; height, 165.2 ± 10.7 cm; weight, 63.1 ± 12.17 kg). The research was conducted at the Department of Radiology, Okubo Hospital (Akashi, Hyogo, Japan) between March 2015 and December 2020. Magnetic resonance imaging (MRI) was performed on both lower legs in a noncooled resting state. The right lower leg was cooled for 15 minutes using an ice bath, then both legs were examined by MRI (Experiment 1). After two weeks, the left lower leg was cooled, and MRI was performed on both legs (Experiment 2). After the subsequent two weeks, MRI was performed on both legs without cooling (Experiment 3). The target areas were subcutaneous and adipose tissues, muscle, bone, and cartilage. T_1_ signal intensity changes after cooling were examined for each tissue. Normality was confirmed by the Shapiro-Wilk test in advance, and the effect size (Cohen's d) was calculated as a post-test when a significant difference was found.

Results: In Experiments 1 and 2, T_1_ signal intensities in subcutaneous tissue, lateral inframalleolar fat pad, the extensor digitorum longus, and abductor hallucis muscles were significantly higher in the cooled than in the noncooled leg (P < 0.05). No significant differences were observed in tissues on the noncooled side.

Conclusions: A 15-minute cold stimulation, such as that used for ankle sprains, reduced temperatures in subcutaneous adipose tissue, muscles, and the lateral inframalleolar fat pad. As the lateral inframalleolar fat pad was effectively cooled, the joint capsule and ligaments immediately below may have also been cooled. It is important to consider the tissue intended for cooling when performing cryotherapy. An ice bath below the lower leg is effective for promoting the recovery of damaged tissue.

## Introduction

Cryotherapy involves conduction cooling (ice and an ice bag), convection cooling (ice water), or vaporization cooling (fluoromethane gas). These methods cool the targeted body area surface, providing pain relief and reducing both swelling and spasticity, which promotes early recovery. Preliminary evidence suggests that intermittent cryotherapy use effectively reduces tissue temperatures to optimal therapeutic levels. Previous studies on humans, dogs, and cats demonstrated that cold stimulation of joints decreased their internal temperature [1-8].

However, Horvath reported that upon cold pack application to humans, though skin surface temperature decreased, there was an increase in intra-articular temperature [9]. These contradictory findings of both increases and decreases in intra-articular temperature require further exploration. As limited information is currently available on the effects of cooling on intra-articular temperatures in the human body, we examined the effects of cooling on various tissues, including muscles.

Magnetic resonance imaging (MRI) provides excellent soft tissue visibility, allowing the measurement of temperature changes in deep tissues based on signal intensities. Herein, we examined temperature change differences between an ice bath cooled lower leg and a noncooled leg by MRI to elucidate the effects of cooling on temperature changes in deep tissues surrounding the ankle joint.

## Materials and methods

Subjects

Seven healthy subjects (four males and three females) were enrolled (age, 21.5 ± 0.8 years; height, 165.2 ± 10.7 cm; weight, 63.1 ± 12.17 kg). The study participants were research volunteers who were given oral explanations in advance. They gave consent regarding the purpose, method, safety considerations, and dangers of the experiment. This study was approved by the Ethics Committee of Okubo Hospital (approval no. 2804).

Target tissues in the present study were as follows: subcutaneous tissue 5.0-7.5 cm proximal to the medial malleolus, adipose tissue below the lateral malleolus, bone tissue on the line connecting the medial malleolus and lateral malleolus, articular cartilage on the underside of the tibia, abductor hallucis and extensor digitorum longus muscles. To identify the cooled target by MRI, Adalat tablets were fixed to the skin as markers of the boundary between cooled and noncooled areas. Subjects rested barefoot for 20 minutes before each experiment.

In the group sessions, the measurement method was explained verbally and with illustrations for about 10 minutes to facilitate understanding. Specifically, after arriving at the facility, the subjects rested barefoot for 20 minutes. Then, both lower legs underwent MRI measurement, and one leg was cooled for 15 minutes. Immediately after that, both lower legs underwent MRI measurement. Since the MRI machine installed in the hospital for medical treatment was used for measurement, only one person could be measured at a time, with a measurement period of 4-6 weeks or longer required for each subject.

**Figure 1:**
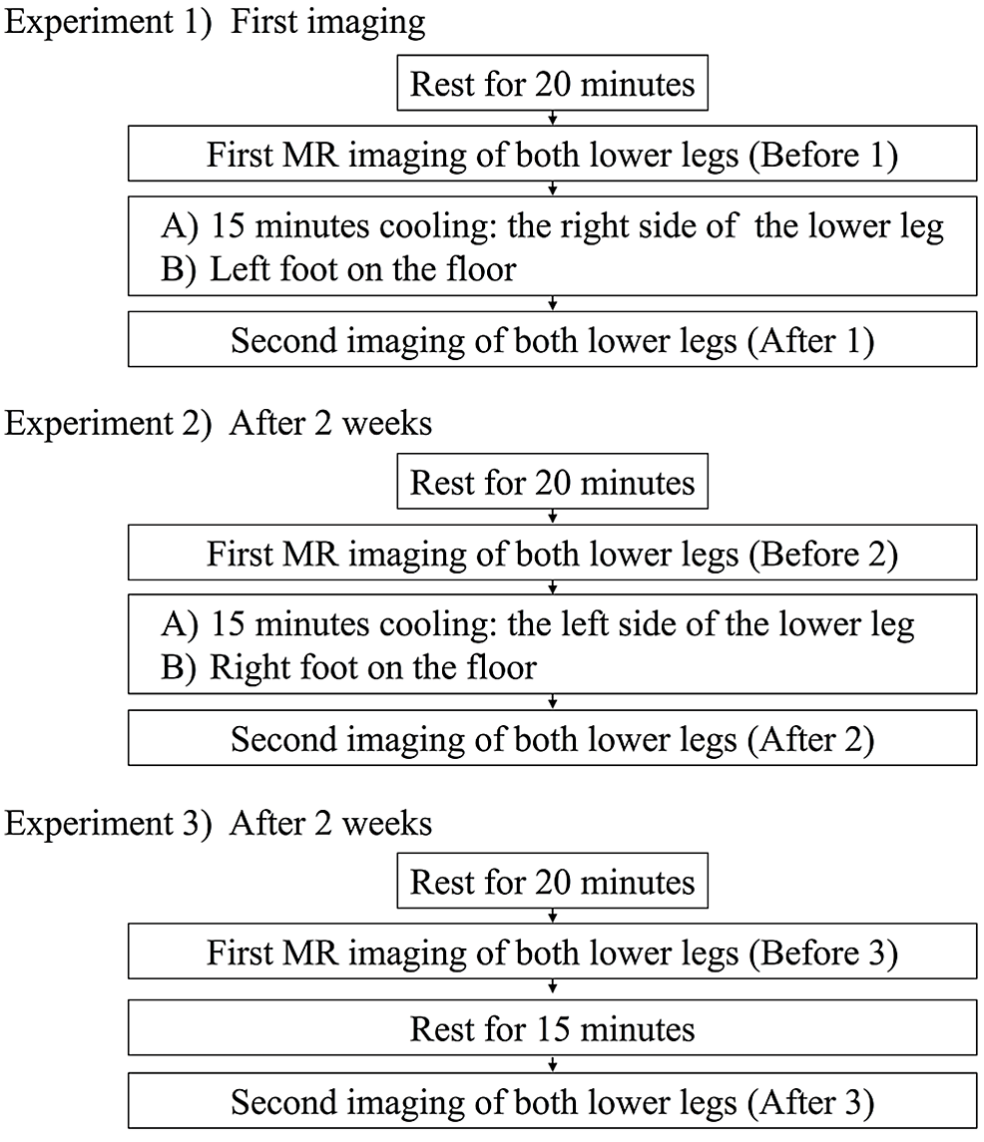
Cooling and MRI protocols

T_1_ signal intensities of the target tissues on T_1_-weighted images for both lower legs and feet were then measured. As controls, MRI was performed on both lower limbs before cooling and on the noncooled side (Figure 1).

In Experiment 1, the lower right leg was submerged in an ice bath containing approximately 3.0 kg of ice and 5 liters of water for 15 minutes. The circumference of the lower leg was about 33.0-38.5 cm. T_1_ signal intensities in both legs were then measured. The ice bath temperature was 1.8-4.2°C. T_1 _signal intensities were defined as precooling/post-cooling. After two weeks, the left lower leg was cooled, and the same measurements were performed as in Experiment 1 (Experiment 2). After two weeks, measurements with 15 minutes resting without cooling were performed (Experiment 3). Surface body temperatures on the central part of the anterior lower leg (approximately 5.0 cm proximal to the lateral malleolus level), anterior ankle joint, and dorsal foot were simultaneously measured with a noncontact infrared thermometer before and after cooling. Changes in pain sensitivity after the cooling stimulus were measured on the Numerical Rating Scale of pain (NRS) from 1 to 10 using a pain evaluation roulette device.

MRI measurements

MRI temperature parameters include the T_1_ relaxation time, T_2_ relaxation time, water molecule diffusion coefficient, and proton chemical shift. Therefore, T_1_- and T_2_-weighted images, diffusion-weighted images, phase images, MR spectroscopy, and proton reference frequency are suitable sequences for temperature measurements.

MRI was performed in the present study using a TOSHIBA Vantage Titan with a magnetic field strength of 1.5 T. The sequence used for imaging was the fast spin echo method, the imaging method was T_1_-weighted images, with a repetition time of 250 msec, an echo time of 20 msec, and a matrix size of 256 × 256. Five consecutive coronal sections with a 5-mm thickness were imaged, and the mean T_1_ signal intensity was calculated from three dorsal side images.

The region of interest was a circle with an area of 0.35 cm^2^ and the field of view was 32 × 45 cm. The temperature of the room in the MRI facility was approximately 25°C and the humidity was 28%-55%.

Evaluation of temperature changes based on the T_1_ relaxation time

The temperature dependence of the T_1_ relaxation time increases with temperature and is expressed by the following equation.

$T_{1}\propto e^{-E_{\alpha }\left ( T_{1} \right )/kT},$

Where Eα(T_1_) is the activation energy of the relaxation process, k is the Boltzmann constant, and T is the absolute temperature. Within a small temperature, the range T_1_ is linearly dependent on the temperature [10].

Statistical analysis

R (ver.4.1.2) was used for statistical analysis, to compare the cooled and noncooled T_1 _signal values by the paired t-test. The significance was set at 5%. Normality was confirmed by the Shapiro-Wilk test in advance, and the effect size (Cohen's d) was calculated as a post-test when a significant difference was found. In cases where normality was not rejected, the t-test was used, and in cases where normality was rejected, the Mann-Whitney U test evaluated the similarity between left and right-side distributions.

Before cooling at rest in all experiments, normality was significantly rejected in the right subcutaneous tissue, right and left body fat, right and left tibias, right and left flexor muscles, and abductor right and left muscles. As a result, no significant difference was found in any experiments.

After cooling in Experiment 1, normality was significantly rejected in the left fat pad, left tibia, right and left cartilage, right and left flexor muscles, and left abductor muscle. As a result, significant differences were observed among the subcutaneous tissue, fat pad, flexor muscle, and abductor muscle.

After cooling in Experiment 2, normality was significantly rejected on the left tibia, left cartilage, and right abductor muscle. As a result, significant differences were observed among the subcutaneous tissue, fat pad, flexor muscle, and abductor muscle. Cohen’s d showed significantly large size effects in these tissues. Before cooling, no significant differences were observed between the left and right sides in any experiment.

## Results

The mean axillary temperature was 36.48 ± 0.039. The mean skin surface temperature at six sites on the left and right legs at rest before cooling was approximately 32.0°C. The surface temperature of the cooled leg in Experiments 1 and 2 decreased by approximately 20°C, whereas the non-cooled side decreased by about 1°C (Table 1).

**Table 1:** Skin surface temperatures Values are mean ± standard deviation

	Center of the lower leg		Center of the ankle		Center of the dorsal foot	
Experiment 1	Cooled side (right)	Non-cooled side (left)	Cooled side (right)	Non-cooled side (left)	Cooled side (right)	Non-cooled side (left)
Pre-cooling 1	32.30 ± 1.30	31.62 ± 1.77	32.25 ± 1.36	32.32 ± 1.25	32.29 ± 1.30	32.26 ± 1.31
Post-cooling 1	13.90 ± 1.01	33.71 ± 0.75	13.59 ± 0.91	33.0 ± 1.43	13.16 ± 1.08	33.32 ± 1.31
Experiment 2	Cooled side (right)	Non-cooled side (left)	Cooled side (right)	Non-cooled side (left)	Cooled side (right)	Non-cooled side (left)
Pre-cooling 2	32.37 ± 1.66	32.16 ± 1.73	32.36 ± 1.62	32.39 ± 1.60	32.47 ± 1.66	32.44 ± 1.63
Post-cooling 2	13.07 ± 0.82	33.58 ± 0.73	12.77 ± 0.94	33.27 ± 1.25	12.67 ± 0.94	33.24 ± 1.55

No significant differences were observed in the T_1_ signal intensities in tissues in both legs before cooling in Experiment 1 or 2 (pre-cooling 1 and pre-cooling 2, respectively, in Tables 2, 3). T_1_ signal intensities in subcutaneous tissue, the lateral inframalleolar fat pad, and extensor digitorum longus and abductor hallucis muscles were significantly elevated after cooling compared with before cooling in both Experiments 1 and 2 (Tables 2, 3).

**Table 2:** Experiment 1: T1 signal strength before and after cooling

	Experiment 1											
	Subcutaneous tissue		Lateral inframalleolar fat pad		Tibia (bone)		Cartilage on the underside of the tibia		Extensor digitorum longus		Abductor hallucis	
	Resting before cooling (Before 1)											
	Right	Left	Right	Left	Right	Left	Right	Left	Right	Left	Right	Left
Mean ± SD	1026 ± 110.16	1033 ± 113.26	763 ± 135.21	758.54 ± 118.13	964.16 ± 169.13	982.32 ± 179.6	86.95 ± 25.74	88.29 ± 23.62	192 ± 49.81	196.15 ± 52.48	204.83 ± 54.32	203.45 ± 50.06
Mean difference	−7.30		4.57		−18.17		−1.34		−3.31		1.38	
Shapiro–Wilk test (p-value)	0.0371	0.0581	0.0012	0.0145	0.0011	0.0015	0.4186	0.3984	0.0000	0.0000	0.0001	0.0002
T test (p-value)	0.3134		0.6761		0.2746		0.5926		0.1145		0.5628	
Cohen's d	0.0638		0.0351		0.1016		0.0528		0.0632		0.0258	
Effect size	Negligible		Negligible		Negligible		Negligible		Negligible		Negligible	
Mann– Whitney U test (p-value)	0.8014		0.9010		0.7462		0.8617		0.5667		1.0000	
	After cooling the right foot (After 1)											
	Cooling (right)	No cooling (left)	Cooling (right)	No cooling (left)	Cooling (right)	No cooling (left)	Cooling (right)	No cooling (left)	Cooling (right)	No cooling (left)	Cooling (right)	No cooling (left)
Mean ± SD	1080.68 ± 142.06	979.81 ± 110.78	794.53 ± 148.66	719.34 ± 117.68	965.54 ± 172.33	969 ± 178.44	92.90 ± 23.48	88.52 ± 24.51	211.81 ± 46.48	184.71 ± 36.63	236.20 ± 51.88	199.56 ± 53.04
Mean difference	100.87		75.19		-3.95		4.38		27.10		36.64	
Shapiro-Wilk test (p-value)	0.3484	0.3951	0.2370	0.0102	0.2697	0.0281	0.0138	0.0221	0.0159	0.0627	0.2248	0.0065
T test (p-value)	0.0000		0.0000		0.7409		0.1498		0.0000		0.0000	
Cohen's d	0.7728		0.5473		0.0220		0.1779		0.6321		0.6816	
Effect size	Medium		Medium		Negligible		Negligible		Medium		Medium	
Mann - Whitney U test (p-value)	0.0336		0.0062		0.9010		0.3923		0.0295		0.0073	

**Table 3:** Experiment 2: T1 signal strength before and after cooling

	Subcutaneous tissue		Lateral inframalleolar fat pad		Tibia (bone)		Cartilage on the underside of the tibia		Extensor digitorum longus		Abductor hallucis	
	Resting before cooling (Before 2)											
	Right	Left	Right	Left	Right	Left	Right	Left	Right	Left	Right	Left
Mean ± SD	1027.34 ± 96	1037.99 ± 116.93	739.15 ± 97.91	744.56 ± 127.53	957.55 ± 163.02	968.30 ± 158.42	72.47 ± 32.89	74.10 ± 38.28	186.77 ± 22.35	188.79 ± 16.59	200.06 ± 30.14	198.94 ± 30.28
Mean difference	−10.65		−5.41		−10.75		−1.62		−2.02		1.13	
Shapiro–Wilk test (p-value)	0.1792	0.1835	0.3760	0.3498	0.0037	0.002135	0.1508	0.0323	0.0882	0.1914	0.2068	0.1209
T test (p-value)	0.4165		0.6575		0.4434		0.6514		0.4290		0.7328	
Cohen's d	0.0972		0.0464		0.0653		0.0444		0.0999		0.0364	
Effect size	Negligible		Negligible		Negligible		Negligible		Negligible		Negligible	
Mann - Whitney U test (p-value)	0.7275		0.8617		0.6904		0.9405		0.7842		0.8422	
	After cooling the right foot (After 2)											
	Cooling (right)	No cooling (left)	Cooling (right)	No cooling (left)	Cooling (right)	No cooling (left)	Cooling (right)	No cooling (left)	Cooling (right)	No cooling (left)	Cooling (right)	No cooling (left)
Mean ± SD	1227.18 ± 110.34	969.34 ± 45.65	837.82 ± 111.99	712.18 ± 98.89	989.73 ± 155.74	992.62 ± 159.72	78.10 ± 31.69	79.70 ± 41.71	182.92 ± 27.77	227.03 ± 26.31	208.01 ± 46.27	235.53 ± 21.30
Mean difference	257.84		116.63		−2.89		−1.60		−44.11		−27.52	
Shapiro–Wilk test (p-value)	0.1079	0.4230	0.0964	0.1886	0.7057	0.0120	0.1653	0.0076	0.4539	0.5006	0.0007	0.8673
T test (p-value)	0.0000		0.0000		0.8342		0.7620		0.0000		0.0093	
Cohen's d	2.9800		1.0774		0.0179		0.0421		1.5915		0.7456	
Effect size	Large		Large		Negligible		Negligible		Large		Medium	
Mann– Whitney U test (p-value)	0.0000		0.0012		0.9010		0.4539		0.0000		0.0010	

No significant differences were observed in the T_1_ signal intensities of tissues in both legs before cooling in Experiment 1 or 2 (pre-cooling 1 and pre-cooling 2, respectively, in Tables 2, 3). T_1_ signal intensities in subcutaneous tissue, the lateral inframalleolar fat pad, and extensor digitorum longus and abductor hallucis muscles were significantly elevated after cooling compared with before cooling in both Experiments 1 and 2 (Tables 2, 3).

No significant changes were observed in the tissues on the noncooled side in Experiments 1 and 2, or in the noncooled lower limbs after resting for 15 minutes in Experiment 3 (Table 4).

**Table 4:** Experiment 3: T1 signal strength after a 15-minute rest

	Subcutaneous tissue		Lateral inframalleolar fat pad		Tibia (bone)		Cartilage on the underside of the tibia		Extensor digitorum longus		Abductor hallucis	
	Before resting (Before 3)											
	Right	Left	Right	Left	Right	Left	Right	Left	Right	Left	Right	Left
Mean ± SD	1029.55 ± 114.27	1028.97 ± 85.68	865.43 ± 105.97	870.66 ± 122.22	1034.27 ± 116.00	10591.73 ± 84.73	89.34 ± 21.13	85.42 ± 24.44	201.24 ± 51.57	193.02 ± 37.43	227.84 ± 59.06	222.43 ± 40.47
Mean difference	0.58		−5.23		−25.46		3.91		8.22		5.42	
Shapiro–Wilk test (p-value)	0.02292	0.4074	0.7807	0.364	0.05109	0.3946	0.07906	0.02666	0.0978	0.5114	0.05082	0.2408
T test (p-value)	0.9815		0.7827		0.2213		0.1705		0.1446		0.5366	
Cohen's d	0.005573731		−0.04463965		−0.2446001		0.1670672		0.1780801		0.1043971	
Effect size	Negligible		Negligible		Small		Negligible		Negligible		Negligible	
Mann–Whitney U test (p-value)	0.8813		0.8813		0.2399			0.3391		0.8228	0.8617	
	After a 15-minute rest (After 3)											
	Cooling (right)	No cooling (left)	Cooling (right)	No cooling (left)	Cooling (right	No cooling (left)	Cooling (right)	No cooling (left)	Cooling (right)	No cooling (left)	Cooling (right)	No cooling (left)
Mean ± SD	1062.80 ± 103.23	1080 ± 107.09	921.55 ± 125.29	910.78 ± 146.84	1037.40 ± 118.64	1062.38 ± 128.65	83.92 ± 17.47	89.01 ± 19.63	190.58 ± 27.10	187.60 ± 32.08	213.30 ± 52.75	209 ± 48.72
Mean difference	-17.52		10.77		-24.99		-5.09		2.98		3.92	
Shapiro- Wilk test (p-value)	0.682	0.3242	0.4723	0.851	0.03357	0.05222	0.9379	0.9778	0.239	0.305	0.002	0.003
T test (p-value)	0.4982		0.6483		0.3292		0.1588		0.5423		0.4571	
Cohen's d	−0.1625596		0.07698071		−0.1970634		−0.2673158		0.09805097		0.07528159	
Effect size	Negligible		Negligible		Negligible		Small		Negligible		Negligible	
Mann– Whitney U test (p-value)	0.6184		0.8813		0.4104		0.4395		0.7842		0.9207	

The NRS for determining pain sensation changes after cooling was close to 0 in both Experiments 1 and 2 (Table 5). NRS on all noncooled sides was 10.

**Table 5:** Changes in pain after cooling by NRS

	Experiment 1		
	Center in front of lower leg (in ice water)	Center in front of ankle	Center of foot back
	Cooling (right)	Cooling (right)	Cooling (right)
Maximum	1	1	1
Minimum	0	0	0
Average	0.44	0.22	0.33
	Experiment 2		
	Cooling (left)	Cooling (left)	Cooling (left)
Maximum	2	1	1
Minimum	0	0	0
Average	0.78	0.33	0.22

## Discussion

Accurately monitoring temperature changes in vivo and a detailed understanding of their effects are important for the proper application of 15-minute cryotherapy. In the present study, the surface temperature of the cooled area decreased by approximately 20°C accompanied by significantly increased T_1_ signal intensities in subcutaneous tissue, the lateral inframalleolar fat pad, and the extensor digitorum longus and abductor hallucis muscles.

This study used the fast spin echo method to image tissues, requiring less than 2 seconds to image each frame, with near real-time data obtained. As ligament water content the around the ankle joint is low, clear visualization is difficult due to the requirements of T_1_-weighted images.

A linear relationship exists between T_1_ relaxation times and temperature. The slope of this relationship varies depending on the tissue type and was previously reported to range between approximately 5-12 ms/°C [11-14].

It is important to note that T1 signal intensity is a relative value due to the characteristics of MRI in which images are constructed to improve visualization. Therefore, in the noncooled legs in Experiments 1, 2, and 3, T_1_ signal intensities may differ even in the absence of a significant change in skin surface temperature. However, T_1 _signal intensities may be compared when the left and right lower legs are simultaneously imaged on the same screen.

Regarding changes in the T_1_ signal intensity of each target tissue, it is estimated that the temperature decreased by 13.6°C or more in the subcutaneous tissue, 11.0°C or more in the lateral ankle fat pad, 4.2°C or more in the extensor digitorum longus muscle, and 4.1°C or more in the abductor hallucis muscle. The temperature of the skin surface was 13 to 27°C lower than the original body temperature, and the skin temperature was reported to be 9.5 to 15.0°C, supporting our findings [3,15-18].

According to Van’t Hoff’s law, a 10% decrease in body temperature results in a 50% decrease in the rate of metabolism of body tissues. Therefore, the T_1_ signal intensities of the subcutaneous adipose tissue and the lateral sub-ankle fat pad decreased by more than 50%, and those of the extensor digitorum longus and abductor hallucis muscle decreased by about 20%, indicating a decrease in metabolism. On the other hand, cooling the lower leg with an ice bath for 15 minutes did not result in an increase in T_1_ signal intensity for the central tibia or subtibial cartilage. Therefore, no significant changes were observed in intra-articular temperature with cooling.

The thermal conductivity and body tissue-specific heat are as follows: 2.78 (cal/sec °C × 103) and 0.38 (cal/g °C) for bone, 1.53 and 0.895 for muscle, 1.31 and 0.87 for blood, 0.898 and 0.9 for skin, and 0.45 and 0.55 for subcutaneous adipose tissue [19]. Thermal conductivity is higher in the order of bone, muscle, blood, skin, and subcutaneous fat, indicating differences in heat transfer even as a temperature stimulus is applied. Therefore, there were temperature differences (T_1_ values) among sites cooled at the same temperature (Tables 2, 3).

In the present study, body surface cooling may transfer arterial blood warmth through veins and arteriovenous anastomosis. The specific heat of body tissue is higher in the order of skin, muscle, blood, subcutaneous adipose tissue, and bone. The cold stimulus cooling effects used in this study showed that distal bone and ankle joint cartilage were not significantly affected by the cold stimulus in terms of T1 signal intensity.

When body temperature is decreased by 10°C, the metabolic rate is halved, which suppresses oxygen activity and reduces collagen fiber degeneration [20,21]. This represents an effective approach for reducing secondary damage. On the other hand, a temperature of 42°C has been shown to promote macrophage migration and satellite cell differentiation. Further studies are needed to examine the effects of cold stimulation on skeletal muscle recovery [22-25]. When skin temperature decreases below 20°C, pain impulse conduction decreases, and γ nerve fibers are suppressed, resulting in decreased muscle tone [26,27].

After ice bath cooling for 15 minutes in Experiments 1 and 2, the mean NRS of the lower leg, ankle, and instep were 0.14 and 0.71, respectively, showing a marked reduction in pain sensation (Table 5). This suggests that ice bath cooling sufficiently reduces pain impulse conduction. Thus, longer cooling times may increase the effects of lowering the pain sensation, but sufficient pain-relieving effects can be obtained even with cooling for 15 minutes.

This study has some limitations. First, the effects of heat generation by water molecules induced by the RF pulse wave during MRI measurements with a magnetic field strength of 3.0 T represent an increase of approximately 0.5°C over 200 seconds. In this study, however, the inductive heating effect of MRI in 1.5 T over 120 seconds was negligible compared to normal body temperature changes.

## Conclusions

As the lateral inframalleolar fat pad was sufficiently cooled, the joint capsule and ligaments immediately below are also likely to be cooled. We conclude that a 15-minute ice bath on the lower leg does not affect the bone marrow or cartilage in the joints, but it can lower the temperature of many other deep body tissues. This method has been found to be effective in the early treatment of many soft tissue injuries. When performing cryotherapy, it is important to carefully consider the tissue intended for cooling. In the future, it will be necessary to improve consistency between direct temperature measurements in living tissues and noninvasive temperature measurements using MRI.
